# Muscle Activation during Gait in Unilateral Transtibial Amputee Patients with Prosthesis: The Influence of the Insole Material Density

**DOI:** 10.3390/jcm10143119

**Published:** 2021-07-15

**Authors:** Nuria Sarroca, María José Luesma, José Valero, Javier Deus, Josefa Casanova, Manuel Lahoz

**Affiliations:** 1 Private Practice, Madre Vedruna 14 Bajo Derecho, 50008 Zaragoza, Spain; nuriasarroca@hotmail.com; 2 Department of Human Anatomy and Histology, University of Zaragoza, 50009 Zaragoza, Spain; mlahozg@unizar.es; 3 Private Practice, Coso 55, 50001 Zaragoza, Spain; clinicajosevalero@gmail.com; 4 Department of Surgery, University Hospital, 50009 Zaragoza, Spain; jdeus@unizar.es; 5 Checa Health Centre, 19310 Guadalajara, Spain; fitacasanova@hotmail.com

**Keywords:** transtibial amputation, electromyography, plantar orthosis

## Abstract

Background: Walking is a complex process that is highly automated and efficient. This knowledge is essential for the study of pathological gait. The amputation of lower limbs involves new biomechanical load and gait patterns, and injuries due to overload or disuse may occur. The objective of this study is to assess muscle activation as part of the gait in unilateral transtibial amputee patients with prosthesis, at different speeds and with different plantar supports. Method: Included in the sample were 25 people with amputation and 25 control participants. Muscle activation was evaluated in both groups by means of surface electromyography (EMG) under normal and altered conditions. Results: Control participants did not show statistically significant differences (*p* ˃ 0.05) between their muscle groups, irrespective of support and speed. However, people with amputation did show differences in muscle activity in the quadriceps, all of which occurred at the highest speeds, irrespective of support. In the analysis between groups, significant differences (*p* < 0.05) were obtained between the leg of the amputee patient and the leg of the control participant, all of them in the quadriceps, and at speeds 3 and 4, regardless of the insole used. Conclusions: Participants with unilateral transtibial amputation carry out more quadriceps muscle activity during gait compared to the control group.

## 1. Introduction

The gait is the mode of locomotion whereby the subject never leaves the ground and maintains a dynamic balance. When a person walks, the body can be assimilated to a mass that is subject to translational movement, and it is subject to the action of gravity, inertia and acceleration. During this movement, resistance forces that cause an expenditure of energy must be overcome [1,2].

The forces that the body needs to propel itself forward, in the case of a below-knee amputee, are mainly generated by the thigh muscles [3,4]. Of particular importance are the compensatory mechanisms that are necessary for body support and forward propulsion due to the loss of the plantar ankle flexors [3,5,6,7].

Since the electromyography (EMG) signal reveals muscle activity, it is also very useful when studying the movement and coordination of the muscles of the human body, as well as its mechanisms, when performing highly complex tasks, such as the act of walking [8,9].

Although the myoelectric activity of people with transtibial amputation could be expected to differ from that of control individuals, due to differences in their overall kinetics [10,11], some groups have reported similar EMG patterns of the knee musculature in the amputated limb to those of the intact limbs in control individuals [12,13,14].

There is a limited understanding of how speed and the use of different plantar supports measured by myoelectric signals can affect people with amputation. The possibility of an increase in magnitude of the activation of knee muscles in the amputated and contralateral limbs of transtibial amputee individuals, with the demands of added speed, has been reported [13,15].

The aim of our study to analyse and measure the influence of insoles made of soft and rigid material on the instability of transtibial amputee patients with prostheses versus control group, by using treadmills at four speeds and in different situations (barefoot, footwear without insoles, footwear with soft insoles, and footwear with rigid insoles).

The study could contribute to a better understanding of the biomechanical gait pattern in subjects with unilateral transtibial amputation that would enable us to identify what motor strategies they would adopt to replace the lack of a biological limb during gait.

## 2. Methods

### 2.1. Design

A prospective pre-post longitudinal quasi-experimental study was carried out.

The protocol was verified and approved by the Research Ethics Committee of the Community of Aragon (CEICA) (Registration no: PI18/403), and all subjects signed an informed consent form prior to participating in the study.

The ethical principles for medical research involving human subjects of the Declaration of Helsinki adopted at the 18th Assembly of the World Medical Association (WMA) (Helsinki, Finland, June 1964) were followed, including amendments made at the 52nd General Assembly (Edinburgh, Scotland, October 2000), with clarification note of paragraph 29 (General Assembly of the WMA, Tokyo 2004) and last revised version at the 59th General Assembly of the WMA, held in Seoul (Korea, October 2008) [16].

### 2.2. Participants

Two population groups were recruited through voluntary participation.

The first group comprised 25 unilateral transtibial amputee participants between 18 and 70 years old with prosthesis. The inclusion criteria were that all subjects were free from musculoskeletal disorders and leg pain, they were competent walkers, and could walk without devices. Each patient used his/her own prosthesis, and had at least two years’ experience with the device. The amputee participants did not have knee instability. The alignment of each prosthesis was checked by a technician before the test. All subjects used axial type prosthetic feet, and gave informed consent to the study.

The second group comprised 25 control participants (control group) with no stability problems in standing position or mobility problems, and who gave their consent to the study. These were non-amputee participants between 18 and 70 years old, with similar height and weight to those of the amputee participants, and whose medical histories did not indicate any osteoarticular or neurological disorders.

The control participants were recruited from the Sarroca Podiatry Clinic, and the transtibial amputee individuals came from several entities: Asociación de Amputados Adampi, Ortopedia Axis, Ortopedia Alcalá, Ortopedia Zaraorto, and different hospitals according to a random sampling method, after identifying the eligible population through a clinical examination by the principal investigator to determine inclusion.

### 2.3. Electromyographic Study

The electromyographic study was conducted by the same clinician who performed all measurements for all study participants.

The gait data were collected at the Sarroca Podiatry Clinic and tabulated into a database designed for the study. After a short adaptation of the participants to the treadmill, it was assessed whether they were able to assume four different speeds (V1 = 0.7, V2 = 1.0, V3 = 1.3, and V4 = 1.6 m/s) on it.

After that, the individuals stepped on the automated treadmill to carry out the study (NordicTrack T12.2, Model NETL 12812.0, Icon (Health&Fitness, Logan, UT, USA) [17]. The participants walked for two minutes at each of the four speeds [18].

In order to classify the individuals’ adaptation to the speed, and to be able to safely complete the level of gait, we asked each participant to start walking from the lowest speed. If they could walk comfortably at that speed, we gradually increased it to the next level. We continued in this way until the fastest speed was reached, or until the subject could no longer maintain the speed when walking [18].

The intensity of muscle activation was calculated from EMG, specifically the mean value of rectified and averaged EMG (microvolts = µV) over the entire V1/V2/V3/V4 sequence.

In our study, all amputee individuals used the same type of Vari-Flex^®^ (Össur hf, Reykjavík, Iceland) prosthetic foot with rigid fitting carbon fibre TSB (Total Surface Bearing), and the same trainer model during the test.

EMG was measured with knitted fabric EMG shorts (EMG MBody^®^ from Myontec Ltd., Kuopio, Finland) similar to garments used for sports activities, or functional underwear [19]. EMG measured with Myontech pants has been found to be feasible for studies of muscle activation during gait [19].

The shorts measured EMG of the quadriceps femoris area (vastus lateralis, vastus medialis and vastus cruraeus, and rectus femoris), and hamstring area (biceps femoris, semimembranosus and semitendinosus) [18].

The signals of the thigh muscles of the amputated leg were compared with the signals of the healthy leg muscles, as were the signals in both legs in the control participants. This measurement was intra-group.

In the amputee group, the signals from the thigh muscles of the amputated leg were also compared with the signals from the right thigh muscles in the control individuals. The right side of the body was the reference side used in other studies [11,20,21]. This measurement was inter-group.

Our objective was to evaluate how the intensity of muscle activation varied by applying different situations and insoles at four different speeds. This analysis enables a reliable comparison of data between the control group and amputee participants [22].

The participants’ skin was prepared, in the area of the electrodes, to reduce existing impedance in order to obtain a quality electrical signal. For this purpose, shaving and rubbing the skin with an abrasive gel was recommended to reduce the layer of dry skin or dead cells. Cleaning the skin with alcohol was also recommended to eliminate sweat [23].

To obtain the average rectified EMG value (AEMG), the shorts were equipped with electrodes and conductive cables integrated into the fabric, which transferred the EMG signals from the electrodes to the electronic piece. The textile electrodes were sewn into the interior of the surface of the shorts, consisting of threads that included silver fibres and non-conductive threads of synthetic fabrics to form a fabric strip.

**Figure 1:**
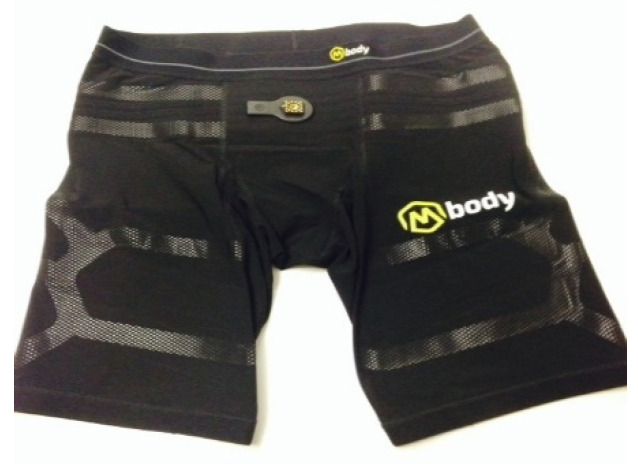
Electromyography (EMG). MBody shorts on the outside.

**Figure 2:**
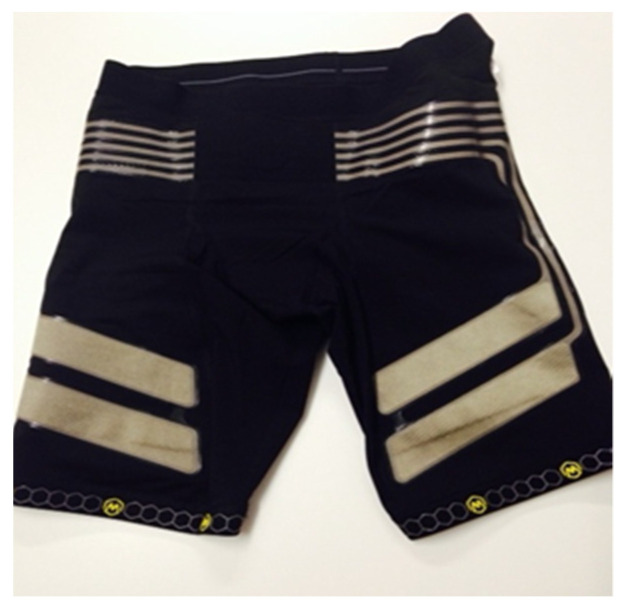
EMG MBody shorts on the inside.

The electrodes were positioned in such a way that the bipolar pair of electrodes was located on the distal side of the thigh electrodes, and the reference electrodes on the lateral sides (Figure 1 and Figure 2) [24].

Minimising the motion artifact was achieved by choosing the right size of shorts (small, medium, large), ensuring that they were tight enough. In addition, because the wires were sewn on the internal side of the shorts, the wire movement was greatly reduced, thus decreasing the likelihood of motion artifacts [24].

The electronic module contained signal amplifiers, an embedded microprocessor with software, data memory, and a PC interface. In the module, the EMG signal was measured in its raw form with a sampling frequency of 1000 Hz, and a frequency band of 50–200 Hz (−3 dB) [24]. These artifacts are very difficult to detect and to remove automatically from the averaged data stored in the module [19].

The bandwidth used for the measurements in the equipment is very reliable for reducing possible high-amplitude motion artifacts that mainly occur at frequencies below 50 Hz [24].

The raw EMG signal was first rectified and then averaged over 100 m intervals. The averaged data were stored in ASCII format in the memory of the module, from which the data were downloaded to a PC using the specifically designed Heimo PC software (Myontec Ltd., Kuopio, Finland) [19].

Prior to data collection, the EMG signals of maximum voluntary contraction (MVC) of each muscle group were recorded. These data were processed and used to normalise the EMG activity of the respective muscle during the dynamic task.

MVC was necessary to normalise the tracings obtained with respect to the maximum activity of that muscle and in that individual. Three maximum isometric contractions of 6 s were obtained with 2 min rest between tests to avoid muscle fatigue [25], and the average of these three measurements was used.

**Figure 3:**
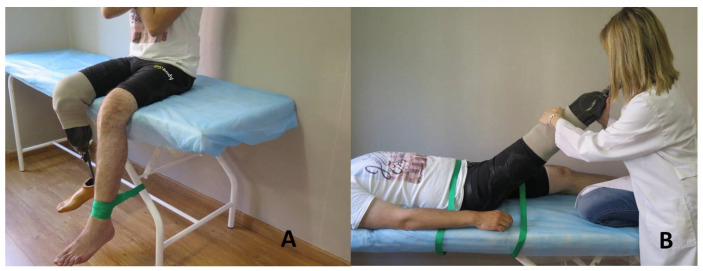
Recording of the maximum voluntary contraction (MVC). (**A**) Quadriceps group MVC record. (**B**) Hamstring group MVC record.

To record the MVC of the quadriceps, the participants were seated on a stretcher with a hip flexion of 90° so that their feet were not in contact with the ground during the voluntary muscle activation tests. These were secured to the stretcher by means of pole therapy straps, to avoid movements and compensation for effort by another muscle group (Figure 3A). To obtain MVC of the hamstring group, a stretch based on the proprioceptive neuromuscular facilitation technique was chosen. The therapist took up a cranial and ipsilateral position with respect to the participant’s limb to be stretched, with one leg flexed on the stretcher. The individual was placed in supine position, flexing the hip of the limb to be stretched, keeping the knee extended and the leg on the therapist’s shoulder. The other limb was extended on the table. The therapist flexed the participant’s hip until he/she reported tension in the hamstring. The subject was then asked to carry out an isometric quadriceps contraction resisted by the therapist’s hand for three seconds. The subject then relaxed the leg for two seconds, and afterwards the therapist performed a maximal hamstring stretch for 10 s [26,27] (Figure 3B).

The order of the different tests in both groups (barefoot, footwear without insoles, footwear with soft insoles, and footwear with hard insoles) was random, and the EMG shorts were kept on throughout the entire electromyographic study.

**Figure 4:**
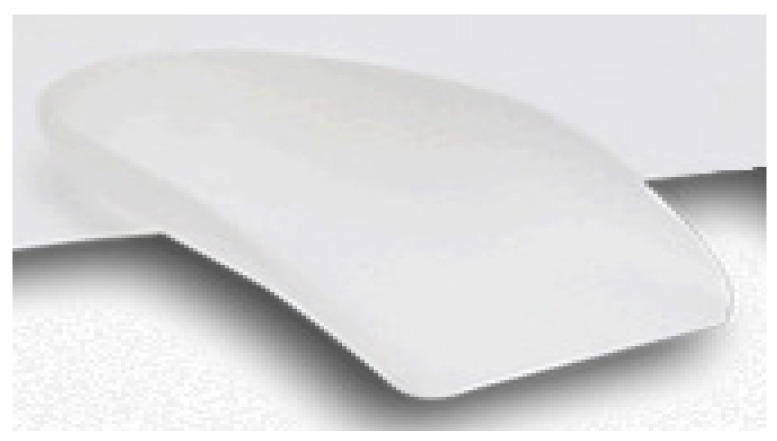
4 mm polypropylene insole PP-DWST.

**Figure 5:**
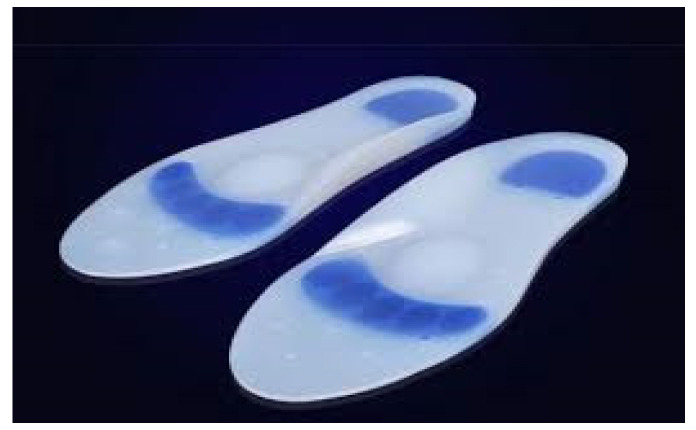
Silicone Comfort Insole.

To examine the influence of the plantar support on the electromyography result, participants carried out the walking tests on four surfaces:

1. Subject with bare feet.
2. Subject wearing running shoes (all with the same model).
3. Subject with running shoes and hard textured surface insole (Figure 4); 4 mm stiff material: Polypropylene PP-DWST. Made by the company SIMONA. (D-55606 Kirn, Germany) and distributed in Spain by Al-Mar Técnicas Ortopédicas S.L. (Arganda del Rey, Madrid, Spain).
4. Subject with running shoe and silicone comfort soft textured surface insole (Figure 5). Soft silicone material. (Varisan© hydrogel insoles, Farmavari S.A.U., Meres, Spain).

### 2.4. Statistical Analysis

Statistical analyses were performed using the statistical software application, IBM SPSS Statistics, version 22 (SPSS Ibérica, Madrid, Spain). 95% confidence intervals were calculated for each variable as well as the mean (SD) (standard deviation).

The Kolmogorov–Smirnov test was used to evaluate the normality of the quantitative variables studied.

The intraclass correlation coefficients (ICC) [28,29] were used to evaluate the reliability of the parameters within the same day of testing in each patient. Using the classification proposed by Landis and Koch [28], ICC between 0.20 and 0.40 were considered to prove reasonable reliability. Scores between 0.40 and 0.60 had moderate reliability, scores between 0.60 and 0.80 had considerable reliability, and in the highest category, scores varying between 0.80 and 1.00 were considered almost perfect. Other authors [30] indicate that to obtain reliability, an ICC value of at least 0.75 must be obtained.

To evaluate the relationship between the study group (amputee participants versus controls), and sociodemographic, clinical and electromyography variables, the Mann–Whitney test or Student’s t test for quantitative variables were used, according to criteria of normality. In the case of qualitative variables, the chi-squared test or Fisher’s test were used by default.

In order to compare electromyography results in the different situations evaluated (speed and support), methods for comparing means for related samples were applied: the Wilcoxon test for two situations, and the Friedman test for three or more when the variable did not follow normal distribution, and Student’s t test or repeated measures ANOVA if the variable followed normal distribution.

The level of significance was set at *p* < 0.05.

## 3. Results

### 3.1. Sociodemographic Characteristics Attending to the Division by Treatment Groups

We had 50 participants in the study; 25 control individuals and 25 amputee participants. In each of the groups, 80.0% of the subjects were men (20/25) and 20.0% women (5/25). The average age of amputee individuals was 44.0 ± 12.9 years and that of controls was 38.4 ± 12.4 years. This difference between the groups was not statistically significant (*p* = 0.124). The average body mass index (BMI) of amputee individuals was 26.4 ± 4.8 kg/m^2^ and that of controls 25.0 ± 3.1 kg/m^2^. This difference between the groups was not statistically significant (*p* = 0.220) Table 1.

### 3.2. EMG Results

To assess the muscle activity, data were provided on the intensity of muscle activation (EMG) in two muscle groups (quadriceps and hamstring), at four walking speeds (V1, V2, V3 and V4), and on four supports (barefoot, footwear without insoles, footwear with soft insoles, and footwear with hard insoles). The study was carried out for both legs of the controls (left leg and right leg), and for both legs of the amputee participants (healthy leg and amputated leg), the leg being the unit of study in this section.

#### 3.2.1. Intra-Group Analysis

The descriptive parameters of muscle activity for the control group, comparing the right leg with the left leg, showed that there were no significant differences (*p* ˃ 0.05) for any of the situations evaluated (data not shown).

The descriptive parameters of muscle activity for the amputee group, comparing the amputated leg with the healthy leg showed that there were six experimental situations in which significant differences were obtained between both legs (*p* < 0.05), all of them in the quadriceps muscle group (barefoot at speed 4, with soft insole at speeds 2, 3 and 4, and with hard insole at speed 4) (Appendix A, Table A1).

#### 3.2.2. Inter-Group Analysis

Table 2 shows the descriptive parameters of muscle activity for controls and amputee participants, comparing the amputees’ leg with the right leg of the controls. Sixteen different situations were considered, at four different walking speeds (V1, V2, V3 and V4), and on four supports (barefoot, no insole, soft insole and hard insole).

In the hamstring muscle group, no statistically significant differences were observed between controls and people with amputations (*p* > 0.05). However, in the quadriceps group, eight experimental situations were observed in which significantly lower average EMG levels were obtained in amputee participants than in controls (*p* < 0.05), all of them at speeds 3 and 4, and regardless of the insole used (the same is true for the four supports).

Both amputee individuals and controls showed statistically significant differences (*p* < 0.05) when the sixteen experimental situations were compared.

The influence of the support was also analysed. Comparisons according to support were studied for the four speeds, for the two muscle groups and for the two study groups. No influence of the support was observed for the lower speeds (V1, V2 and V3); however, at speed 4, it had a significant influence on the hamstring muscle group, both for amputee cases (*p* = 0.032) and controls (*p* = 0.004). When pairwise comparisons were performed, it was observed that “barefoot” was the support that presented significant differences (*p* ˂ 0.05) to the other supports in amputee individuals, and “hard insole” in controls. These conditions displayed significantly higher levels of EMG than the others (Table 3).

The influence of speed was also evaluated. Table 4 shows the comparisons according to speed for the four supports, for the two muscle groups, and for the two study groups. Statistically significant differences were observed in the muscle activity according to the speed used (*p* < 0.05) in all cases. In addition, pairwise comparisons were made, observing significant differences in all pairs.

### 3.3. Reliability Analysis

The measurements showed excellent reliability with CI levels of above 0.90 for all stability variables considered, thus justifying the use of the mean value for the data analysis. The specific CI values are provided in Appendix A, Table A2.

## 4. Discussion

The present study entails finding out how electromyography can easily be put into practice, as well as the dynamics, by including textile electrodes in shorts.

The results and application of this study have provided us with a more in-depth understanding of the movement of the human body based on kinematics in its most complex expression: human gait. It especially contributes to a better understanding of the biomechanical gait pattern in subjects with unilateral transtibial amputation.

The set of variables analysed for thigh EMG, with varying insole hardness and at different speeds, allows us to complete this knowledge. The aim was to obtain a comprehensive perspective of people with unilateral transtibial amputation that would enable us to hypothesise about the possible deviations of their biomechanical pattern, and identify what motor strategies, if any, they would adopt to replace the lack of a biological limb during gait. In addition to effectively managing the alterations, it permitted studying the gait of each amputee patient at an early stage, to be able intervene in an optimal fitting that would compensate their biological limb.

A possible limitation of our study could be that the patients did not walk on the ground, because we wanted to assess the recordings with a constant speed, and this could only be achieved on a treadmill.

Studies have shown that EMG patterns of the lower limbs, and the kinematics, may differ when walking on the treadmill, compared to walking on the ground [31,32]. In contrast to these studies, others point out that biomechanically, walking on a treadmill and walking on the ground are identical if the speed on the treadmill is constant [33].

In 2013, Kawashima et al. [34] assessed the kinaesthesia of the phantom limb of amputee individuals by means of surface electromyography on the muscles of the stump, under the premise that the amputated limb presented electrical-type activity when the person thought or evoked a movement. This study did not take the evaluation of the residual muscles of the stump as a reference, only considering healthy muscles: quadriceps and hamstring, which confer active stability to the knee joint in unipedal stance.

In 2014, Arifin et al. [35] claimed that the loss of the biological ankle joint, and the considerable number of muscles at the distal end of the leg, were associated with reduced proprioception and this, in turn, was associated with asymmetry in weight load, mobility, balance and confidence of amputees.

The results of our study reveal that, in the control population, the muscle groups do not show statistically significant differences, regardless of the support or speed. However, amputee participants, in certain support or speed situations (more unstable support or greater speed) show differences in muscle activity in the quadriceps.

In the amputee group, there were six experimental situations in which significant differences were obtained between the amputated leg and the non-amputated leg. All of these in the quadriceps; when the patient was barefoot, at speed 4, with soft sole at speeds 2, 3 and 4, and with hard sole at speed 4.

In the “Inter” analysis, i.e., comparing the right leg of the control individual with the amputated leg of the case participant, with the four insoles, at the four speeds, and both parts of the leg, eight experimental situations were found in which significant differences were obtained between the leg of the amputee person and the leg of the control individual. All of these were in the quadriceps, and all of them were at speeds 3 and 4, regardless of the insoles used (because the same occurred in the four types of support). No statistically significant differences were observed in the hamstring muscle group.

Regardless of the support (barefoot, no insole, soft insole, or hard insole) and of the muscle group (quadriceps or hamstring), the EMG in our study was significantly altered according to the walking speed; the higher the speed the greater the muscle activity for both amputee individuals and controls.

No influence of the support used was observed for the lower speeds (V1, V2 and V3). However, at speed 4, there was a significant influence on the hamstring muscle group, both for cases and controls.

Previous studies have reported differences in knee muscle activation patterns of transtibial amputee individuals compared to control subjects, specifically in the amount of co-contraction [5,10,13,36].

Other studies have shown greater EMG intensity for all muscles tested in the transtibial amputee group than in the control group, with the highest intensity difference being found in the semimembranosus and biceps femoris [10,12,37,38].

Based on previously published theories, this could be occurring to provide a compensatory stabilising effect, to absorb the extra impact during heel strike [15,39,40]. During this phase, the prosthetic limb is preparing to take off, and generates a lower amount of thrust power (compared to controls), due to the passive nature of the prosthetic foot resulting in a sharper landing for the intact limb [40].

Plantar flexion from the heel to the average support contact is normally possible due to the mobility of the human ankle. However, prosthetic devices do not generally facilitate this function, as they do not have an eccentrically controlled rotational ankle joint [10,12,41].

Our study only shows differences in the hamstring group at speed 4 for both study groups, presenting significantly higher EMG levels in the situation of “barefoot” participants in the amputee group, and with “hard insole” in control participants, compared to the other supports. In future research, we plan to study the use of heel pads that relax the posterior chain to find out if this type of support presents low levels of EMG in the hamstring group in a situation of “shod” patients.

Our results support the use of myoelectric prostheses to favour the advance of robotics in lower limb prostheses, and thus ensure a gait as close as possible to individuals with an intact limb, thus helping to improve their quality of life.

Standardising the prosthetic prescription may be of doubtful efficacy in general terms. People are different, as are their different problems and associated diseases, as well as the shape and length of the residual stump to fit the prosthesis.

However, although from the orthopaedic point of view all amputee people can be fitted with a prosthesis, in practice a satisfactory functional result is not always achieved, and it is necessary to carry out personalised studies to optimise their gait.

This study provides a measure of the muscle activity achieved by the participants with their prosthesis, which can be carried out with different prostheses to assess their optimal performance in their muscle activity with different prosthetic feet.

There are myoelectric prostheses that are electronically controlled by the patients’ voluntary muscle contractions. To advance in the use of these prostheses in the lower limb, it is necessary to carry out studies and provide specific data on EMG values.

Our study yields data on muscle activations in different muscle groups of these patients that can help with different advances in lower limb prostheses.

## 5. Conclusions

In the healthy population, no differences were observed between the muscle groups of both legs, regardless of support and speed. However, subjects with unilateral transtibial amputation showed significantly lower quadriceps muscle activity in the amputated limb than in the healthy leg in certain experimental situations: patient barefoot at speed 4 (1.6 m/s), with hard insoles at speeds 3 (1.3 m/s) and 4 (1.6 m/s), and with soft insoles at speeds 2 (1.0 m/s), 3 (1.3 m/s) and 4 (1.6 m/s).

Lower mean EMG values were obtained in quadriceps when the amputated leg of the experimental group was compared with the right leg of the control group, regardless of the support; the greater the speed, the greater the difference between the groups, reaching statistical significance at speed 3 (1.3 m/s) and speed 4 (1.6 m/s). In both groups, the EMG was significantly altered depending on the walking speed; the greater the speed, the greater the muscular activity, regardless of the support and muscle group.

The support only showed differences in the hamstring group at speed 4 (1.6 m/s) for both study groups, presenting significantly higher EMG levels in the situation of “barefoot” patients in the amputee group, and with “hard insole” in healthy patients compared to the other supports.

## Figures and Tables

**Table 1:** Sociodemographic characteristics of participants according to study group.

Variable	Total *n* = 50	Controls *n* = 25	Amputee Participants *n* = 25	*p* Value *
Male	20 (80.0%)	20 (80.0%)	20 (80.0%)	1.000
Age (years) mean ± SD	41.2 ± 12.9	38.4 ± 12.4	44.0 ± 12.9	0.124
BMI (kg/m^2^) mean ± SD	25.7 ± 4.0	25.0 ± 3.1	26.4 ± 4.8	0.220

BMI, body mass index; SD, standard deviation; * Mann–Whitney U Test.

**Table 2:** EMG according to situation and leg: control right vs. amputees.

	Quadriceps			Hamstring		
	Difference (SD)	%Difference	*p*-Value	Difference (SD)	%Difference	*p*-Value
Barefoot V1	1.2 (6.2)	2.9%	0.977 ^1^	−6.7 (8.0)	−9.5%	0.5281
V2	4.9 (6.7)	9.6%	0.491 ^1^	−6.5 (8.9)	−7.7%	0.4672
V3	13.2 (7.8)	19.2%	**0.049** ^1^	−10.2 (10.2)	−10.2%	0.3212
V4	22.2 (9.1)	24.9%	**0.011** ^1^	−7.6 (11.9)	−6.2%	0.5272
No insole V1	0.3 (5.6)	0.8%	0.946 ^1^	−2.8 (7.0)	−4.0%	0.8311
V2	1.7 (6.3)	3.6%	0.535 ^1^	−4.2 (7.6)	−5.1%	0.5822
V3	11.0 (7.5)	16.8%	**0.030** ^1^	−0.4 (8.4)	−0.4%	0.9672
V4	20.7 (8.8)	24.6%	**0.002** ^1^	5.4 (9.8)	4.5%	0.5882
Soft insole V1	0.3 (4.6)	0.8%	0.473 ^1^	1.6 (8.0)	2.2%	0.6771
V2	7.2 (5.0)	15.1%	0.156 ^2^	1.6 (8.2)	1.8%	0.8472
V3	15.6 (7.0)	24.0%	**0.009** ^1^	1.0 (9.5)	1.0%	0.9202
V4	25.1 (7.8)	29.7%	**<0.001** ^1^	2.5 (11.0)	2.1%	0.8232
Hard insole V1	0.0 (4.9)	0.0%	0.691 ^1^	3.1 (7.8)	4.1%	0.3221
V2	6.5 (5.9)	13.0%	0.221 ^1^	4.1 (8.5)	4.6%	0.5091
V3	16.1 (6.6)	23.8%	**0.013** ^1^	3.1 (10.1)	2.9%	0.7642
V4	24.6 (8.4)	28.5%	**0.002** ^1^	8.6 (11.2)	6.6%	0.4442

Mean (standard deviation) ^1^ Mann–Whitney Test. ^2^ Independent *t* Test for means. V1: speed1, V2: speed 2; V3: speed 3; V4: speed 4. Units: EMG, electromyography); µV, microvolts. Bold values highlight the statistically significant data. Difference = Control − Amputee. %Difference = [(Control − Amputee)/Amputee] * 100.

**Table 3:** EMG for hamstring at speed 4 according to support. Pairwise comparisons (upper diagonal, amputees; lower diagonal, controls).

Hamstring	Barefoot	No Insole	Soft Insole	Hard Insole
Barefoot		**0.001**	**0.009**	0.110
No Insole	0.788		0.840	0.093
Soft Insole	0.893	0.882		0.086
Hard Insole	**0.029**	**0.011**	**0.002**	

Bold values highlight the statistically significant data.

**Table 4:** EMG according to situation and leg: control right versus amputees. Influence of speed.

	Right Leg Controls	Leg Amputees
Barefoot V1 Quadriceps	41.3 (23.1)	40.1 (21.0)
Hamstring	70.8 (23.7)	77.5 (32.4)
V2 Quadriceps	50.9 (24.5)	46.0 (23.2)
Hamstring	84.4 (26.1)	90.9 (35.9)
V3 Quadriceps	68.9 (27.9)	55.7 (27.2)
Hamstring	101.4 (28.7)	111.7 (42.0)
V4 Quadriceps	89.7 (30.5)	67.4 (34.0)
Hamstring	122.9 (33.6)	130.5 (49.2)
*p*-value Quadriceps	**<0.001** ^1^	**<0.001** ^1^
*p*-value Hamstring	**<0.001** ^1^	**<0.001** ^1^
No insole V1 Quadriceps	39.6 (19.6)	39.3 (19.8)
Hamstring	69.6 (18.9)	72.4 (29.3)
V2 Quadriceps	47.2 (19.9)	45.5 (24.7)
Hamstring	82.2 (19.2)	86.4 (32.5)
V3 Quadriceps	65.6 (21.9)	54.6 (30.2)
Hamstring	100.3 (20.8)	100.7 (36.6)
V4 Quadriceps	84.3 (26.6)	63.6 (34.7)
Hamstring	121.2 (26.3)	115.8 (41.6)
*p*-value Quadriceps	**<0.001** ^1^	**<0.001** ^1^
*p*-value Hamstring	**<0.001** ^1^	**<0.001** ^1^
Soft insole V1 Quadriceps	36.3 (19.4)	36.0 (12.1)
Hamstring	74.3 (23.4)	72.7 (32.3)
V2 Quadriceps	47.7 (21.4)	40.5 (13.0)
Hamstring	87.3 (22.3)	85.7 (34.4)
V3 Quadriceps	65.0 (25.9)	49.4 (23.2)
Hamstring	103.6 (21.4)	102.6 (42.5)
V4 Quadriceps	84.5 (26.9)	59.4 (28.5)
Hamstring	121.1 (25.9)	118.6 (48.6)
*p*-value Quadriceps	**<0.001** ^1^	**<0.001** ^1^
*p*-value Hamstring	**<0.001** ^1^	**<0.001** ^1^
Hard insole V1 Quadriceps	37.3 (20.0)	37.3 (14.5)
Hamstring	75.6 (21.0)	72.5 (32.6)
V2 Quadriceps	49.9 (23.3)	43.4 (18.2)
Hamstring	89.6 (25.3)	85.5 (34.0)
V3 Quadriceps	67.6 (24.3)	51.5 (22.2)
Hamstring	106.8 (28.0)	103.7 (41.9)
V4 Quadriceps	86.2 (31.4)	61.6 (27.6)
Hamstring	131.1 (29.8)	122.5 (47.1)
*p*-value Quadriceps	**<0.001** ^1^	**<0.001** ^1^
*p*-value Hamstring	**<0.001** ^1^	**<0.001** ^1^

^1^ Friedman Test. V1: speed1, V2: speed 2; V3: speed 3; V4: speed 4. Units: EMG, electromyography; µV, microvolts. Bold values highlight the statistically significant data.

## Appendix A

**Table A1:** EMG according to situation and leg in the amputee group.

	Healthy Leg	Amputated Leg	Difference	*p*-Value
Barefoot V1 Quadriceps	40.4 (20.2)	40.1 (21.0)	0.3 (20.4)	0.967 ^2^
Hamstring	80.7 (28.2)	77.5 (32.4)	3.2 (29.0)	0.710 ^2^
V2 Quadriceps	51.2 (21.9)	46.0 (23.2)	5.2 (22.7)	0.415 ^2^
Hamstring	92.8 (34.3)	90.9 (35.9)	1.9 (30.4)	0.662 ^1^
V3 Quadriceps	69.5 (26.1)	55.7 (27.2)	13.8 (24.0)	0.074 ^2^
Hamstring	109.6 (42.2)	111.7 (42.0)	-2.1 (42.0)	0.915 ^1^
V4 Quadriceps	86.4 (30.9)	67.4 (34.0)	19.0 (28.7)	**0.044** ^2^
Hamstring	132.6 (52.8)	130.5 (49.2)	2.1 (44.1)	0.938 ^1^
No insole V1 Quadriceps	42.9 (18.5)	39.3 (19.8)	3.6 (20.8)	0.510 ^2^
Hamstring	76.9 (30.1)	72.4 (29.3)	4.5 (33.0)	0.595 ^2^
V2 Quadriceps	52.4 (22.3)	45.5 (24.7)	6.9 (27.2)	0.305 ^2^
Hamstring	90.3 (36.5)	86.4 (32.5)	3.9 (34.2)	0.846 ^1^
V3 Quadriceps	65.1 (23.4)	54.6 (30.2)	10.5 (31.2)	0.175 ^2^
Hamstring	106.5 (43.0)	100.7 (36.6)	5.8 (37.1)	0.923 ^1^
V4 Quadriceps	80.0 (26.1)	63.6 (34.7)	16.4 (33.2)	0.066 ^2^
Hamstring	122.2 (50.4)	115.8 (41.6)	6.4 (39.7)	0.626 ^2^
Soft ins. V1 Quadriceps	44.7 (21.5)	36.0 (12.1)	8.6 (22.2)	0.087 ^2^
Hamstring	77.3 (30.1)	72.7 (32.3)	4.6 (32.3)	0.607 ^2^
V2 Quadriceps	53.1 (23.0)	40.5 (13.0)	12.6 (23.7)	**0.043** ^1^
Hamstring	91.3 (35.5)	85.7 (34.4)	5.6 (33.9)	0.614 ^1^
V3 Quadriceps	67.7 (26.5)	49.4 (23.2)	18.3 (28.3)	**0.012** ^2^
Hamstring	105.7 (44.8)	102.6 (42.5)	3.1 (35.8)	0.915 ^1^
V4 Quadriceps	84.9 (30.1)	59.4 (28.5)	25.5 (34.0)	**0.004** ^2^
Hamstring	123.7 (56.0)	118.6 (48.6)	5.1 (45.3)	0.733 ^2^
Hard ins. V1 Quadriceps	44.7 (17.4)	37.3 (14.5)	7.4 (19.6)	0.107 ^2^
Hamstring	79.5 (31.6)	72.5 (32.6)	7.0 (30.3)	0.440 ^2^
V2 Quadriceps	52.4 (20.3)	43.4 (18.2)	9.0 (24.5)	0.106 ^2^
Hamstring	93.8 (35.4)	85.5 (34.0)	8.3 (28.6)	0.432 ^1^
V3 Quadriceps	69.2 (26.8)	51.5 (22.2)	17.7 (28.7)	**0.015** ^2^
Hamstring	111.8 (44.9)	103.7 (41.9)	8.1 (36.8)	0.778 ^1^
V4 Quadriceps	87.9 (30.5)	61.6 (27.6)	26.3 (33.9)	**0.002** ^2^
Hamstring	128.9 (54.2)	122.5 (47.1)	6.4 (41.0)	0.969 ^1^

Mean (standard deviation) ^1^ Mann–Whitney Test. ^2^ Independent t test for means. V1: speed1, V2: speed 2; V3: speed 3; V4: speed 4. Units: EMG, electromiography; µV, microvolts. Bold values highlight the statistically significant data.

**Table A2:** ICC Reliability Chart (95%) of the three repeated measurements of EMG variables for hamstring.

Variable	ICC	Lower Limit	Limit
HAMSTRING_DS_V1	0.966	0.953	0.976
HAMSTRING_DS_V2	0.949	0.930	0.964
HAMSTRING_DS_V3	0.940	0.917	0.957
HAMSTRING_DS_V4	0.955	0.938	0.968
HAMSTRING_SIN_V1	0.958	0.942	0.970
HAMSTRING_SIN_V2	0.946	0.925	0.962
HAMSTRING_SIN_V3	0.961	0.946	0.972
HAMSTRING_SIN_V4	0.959	0.944	0.971
HAMSTRING_B_V1	0.925	0.898	0.947
HAMSTRING_B_V2	0.947	0.926	0.963
HAMSTRING_B_V3	0.953	0.934	0.967
HAMSTRING_B_V4	0.965	0.952	0.975
HAMSTRING_D_V1	0.955	0.938	0.968
HAMSTRING_D_V2	0.948	0.928	0.963
HAMSTRING_D_V3	0.960	0.943	0.972
HAMSTRING_D_V4	0.967	0.954	0.977

V1: speed1, V2: speed 2; V3: speed 3; V4: speed 4; ICC: intraclass correlation coefficients.
